# Physicochemical and Biochemical Properties of Trypsin-like Enzyme from Two Sturgeon Species

**DOI:** 10.3390/ani13050853

**Published:** 2023-02-26

**Authors:** Abbas Zamani, Maryam Khajavi, Abdolmohammad Abedian Kenari, Masoumeh Haghbin Nazarpak, Atefeh Solouk, Mina Esmaeili, Enric Gisbert

**Affiliations:** 1 Fisheries Department, Faculty of Natural Resources and Environment, Malayer University, 4th km of Arak Road, Malayer 6574184621, Iran; 2 New Technologies Research Center, Amirkabir University of Technology, Tehran 1591634653, Iran; 3 Department of Aquaculture, Faculty of Natural Resources and Marine Sciences, Tarbiat Modares University, Noor P.O. Box 46414-356, Iran; 4 Department of Biomaterial and Tissue Engineering, Medical Engineering Faculty, Amirkabir University of Technology, Tehran 1591634311, Iran; 5 Department of Fisheries, Faculty of Animal Sciences and Fisheries, Sari Agricultural Sciences and Natural Resources University, Sari 4818168984, Iran; 6 IRTA, Centre de la Rápita, Aquaculture Program, Crta. del Poble Nou Km 5.5, 43540 la Rápita, Spain

**Keywords:** beluga, physicochemical properties, *sevruga*, trypsin, digestive physiology

## Abstract

**Simple Summary:**

Beluga and sevruga are *two* highly valuable sturgeon species from the *Acipenseride* family in Iran. In recent years, research has been focused on commercial rearing of these species. A very important aspect in the sturgeon farming industry is the development of formulated compound diets for promoting growth. However, the ability of fish to digest compound diets is mostly related to the existence of the digestive enzymes in different parts of the *gastrointestinal* tract. In gastric species, protein digestion is conducted along the gastrointestinal tract by several proteases such as pepsin, trypsin, and chymotrypsin. Trypsin, as an alkaline protease, is able to hydrolyze protein residues and peptides to release free amino acids and small peptides for intestinal absorption; therefore, the activity of trypsin has been widely used as a valuable indicator of digestive capacity in fish. In this work, we aimed to characterize trypsin from beluga and sevruga for the first time. The results of our study show that the physicochemical and biochemical properties of trypsin from beluga and sevruga were in agreement with data reported in bony fish and may be considered a preliminary step to design in vitro tests for the assessment of protein digestibility in these primitive species.

**Abstract:**

This work aimed to determine the physicochemical and biochemical properties of trypsin from beluga *Huso huso* and sevruga *Acipenser stellatus, two* highly valuable sturgeon species. According to the results obtained from the methods of casein-zymogram and inhibitory activity staining, the molecular weight of trypsin for sevruga and beluga was 27.5 and 29.5 kDa, respectively. Optimum pH and temperature values for both trypsins were recorded at 8.5 and 55 °C by BAPNA (a specific substrate), respectively. The stability of both trypsins was well-preserved at pH values from 6.0 to 11.0 and temperatures up to 50 °C. TLCK and SBTI, two specific trypsin inhibitors, showed a significant inhibitory effect on the enzymatic activity of both trypsins (*p* < 0.05). The enzyme activity was significantly increased in the presence of Ca^+2^ and surfactants and decreased by oxidizing agents, Cu^+2^, Zn^+2^, and Co^+2^ (*p* < 0.05). However, univalent ions Na^+^ and K^+^ did not show any significant effect on the activity of both trypsins (*p* > 0.05). The results of our study show that the properties of trypsin from beluga and sevruga are in agreement with data reported in bony fish and can contribute to the clear understanding of trypsin activity in these primitive species.

## 1. Introduction

Beluga (*Huso huso*) and sevruga (*Acipenser stellatus)* are among the most important species of sturgeon fish (*Acipenseridae*) inhabiting the Caspian Sea with a high demand for products such as caviar, meat, skin, and cartilage [1,2]. Today, sturgeons are considered a vulnerable group of fish species for different reasons such as overfishing for production of meat and caviar, water pollution, and destruction of their natural habitats [3,4]. Therefore, in recent years, researchers have focused their studies on restocking and commercial rearing purposes. According to the Iranian Fisheries Organization report, the aquaculture production of sturgeon has increased from 363 t in 2009 to 2516 t in 2020 [5]. A very important aspect in sturgeon farming industry, affecting its production efficiency and long-term sustainability, is the development of formulated compound diets for promoting growth and product quality. However, the ability of fish to digest compound diets is mostly related to the existence of the digestive enzymes in different parts of the *gastrointestinal* tract [6]. Digestive enzymes reflect the capability of digestion in the organism under study and thus indicate the nutritional status at different stages of growth [7,8]. Herein, analysis of digestive enzymes activity is regarded as a biochemical procedure which may contribute to generate valuable information for understanding the physiology of digestion in fish [9,10]. This important issue can also help to define the requirements of fish for essential nutrients such as proteins, lipids, or carbohydrates [11]. Among macronutrients, dietary proteins are key nutrients for fish growth, since proteins are the building blocks of muscle cells and organs. They occur in a great array of forms, and their nutritional value depends on their amino acid composition. As Moraes and Almeida, 2020 [12] reviewed, the use of dietary proteins depends on a wide array of functional, biochemical, and genetic species-specific characteristics such as the age of organisms; the range of environmental factors (pH, dissolved oxygen, and ammonia levels); the amino acid profile of dietary protein; the digestible usable dietary energy levels; and the presence of antinutritional factors, among others.

Protein digestion is conducted along the gastrointestinal tract by several proteases, with specific actions on the polypeptide chain. In gastric species, pepsin, trypsin, and chymotrypsin are the most important proteolytic enzymes in fish [13,14,15]. As shown by Nolasco-Soria, 2021 [16], trypsin in combination with other alkaline proteases and peptidases such as chymotrypsin, aminopeptidases, and carboxypeptidases complete the acid predigestion conducted in the stomach, hydrolyzing protein residues and peptides to release free amino acids and small peptides for intestinal absorption; therefore, the activity of trypsin has been widely used as a valuable indicator of digestive capacity in fish, as well as a useful biomarker for its nutritional and physiological condition [17]. According to surveys conducted in various species of fish, trypsin participates in activating trypsinogen and other zymogens in the intestine and plays an effective role in protein degradation of the consumed diet in the carnivorous fish up to 40–50% [14,18,19,20,21]. Furthermore, trypsin quantification is essential for the design of in vitro digestibility protocols of feed ingredients and for the formulation of high digestible compound feeds for aquaculture fish species [22].

Hence, a better understanding of the properties of trypsin is necessary to generate valuable information for protein degradation in the fish digestive tract. The characterization of trypsin, especially its physicochemical and biochemical properties, has been thoroughly studied from the intestine of various fish including grass carp (*Ctenopharyngodon idellus*), spotted goatfish (*Pseudupeneus maculatus*), grey triggerfish (*Balistes capriscus*), skipjack tuna (*Katsuwonus pelamis*), smooth hound (*Mustelus mustelus*), and Brazilian flounder (*Paralichthys orbignyanus*) [23,24,25,26,27,28]. The activity of trypsin among several sturgeon species has been mostly studied during larval ontogeny in the members of the genus *Acipenser* such as *A. transmontanus* [29], *A. fulvescens* [30], *A. oxyrinchus* [31,32], *A. baerii* [33], *A. persicus* [34], *A. nacarii* [35,36], *A. stellatus* [37], and genus *Huso* such as *H. huso* [38]. However, there are no studies evaluating the physicochemical and biochemical characteristics of trypsin in sturgeons; thus, this study attempts to characterize trypsin from beluga and sevruga, as two of the main sturgeon species from the Caspian Sea, for the first time.

## 2. Materials and Methods

### 2.1. Fish Samples

Viscera from five specimens of beluga and sevruga (8.0 ± 0.4 kg; 95 ± 8 cm) were obtained from Saei sturgeon rearing center, Sari, Mazandaran, Iran. Fish were fed a commercial diet (crude protein 46%, crude lipid 16%, ash 8.5%, crude fiber 2.5%, and moisture 9%, Mazandaran Animal & Aquatic Feed Company, Semeskandeh Olya, Iran) and kept in fasting condition for 72 h before sampling. Those samples were packed in polyethylene bags, placed in ice with the sample/ice ratio of approximately 1:3 (*w*/*w*), and directly transported to the laboratory. Upon arrival, the intestine was separated from the rest of the collected viscera, washed with cold distilled water (4 °C), pooled, and stored at −80 °C for further analysis.

### 2.2. Preparation of Intestinal Crude Extracts for Trypsin Characterization

The frozen intestine of beluga and sevruga was partially thawed in the refrigerator at 4 °C for 2 h. The samples were then cut into small pieces and homogenized in 50 volumes of 50 mM Tris–HCl buffer (pH 7.5, 10 mM CaCl_2_, 0.5 M NaCl) by a tissue homogenizer (Heidolph Diax 900, Sigma Co., St. Louis, MO, USA) at 4 °C for 2 min. The homogenate was then filtered with a cheese cloth to separate the floating fat phase and centrifuged for 45 min at 4 °C at 14,000× *g* by a refrigerated centrifuge (Hettich Benchtop Centrifuge Rotina 420R, Berlin, Germany). The resulting supernatant from each sample was collected, defined as intestinal crude extract (ICE), and then used throughout this study.

### 2.3. Reagents

EDTA (Ethylenediaminetetraacetic acid), Pepstatin A, PMSF (phenylmethanesulfonyl fluoride), and sodium cholate were obtained from Molekula Co (Gillingham, UK). BAPNA (Nα-benzoyl-DL-arginine-ρ-nitroanilide hydrochloride), ß-mercaptoethanol, BSA (bovine serum albumin), iodoacetic acid, saponin, SBTI (soybean trypsin inhibitor), TLCK (N-ρ-tosyl-L-lysine-chloromethyleketone), and TPCK (N-tosyl-L-phenylalanine chlorom ethyleketone) were purchased from Sigma Chemical Co (St. Louis, MO, USA). Molecular weight marker (PM 2700) was obtained from SMOBIO Technology, Inc. (Hsinchu, Taiwan).

### 2.4. Trypsin Assay

To measure the enzyme activity in ICE, BAPNA was used as a substrate at a concentration of 1 mM in 50 mM Tris–HCl, 20 mM CaCl_2_ (pH 8.5) according to the method of Erlanger et al., 1961 [39]. Each ICE (25 μL) was mixed with the prepared substrate (1250 μL) and incubated at 55 °C for 20 min. The reaction was terminated by adding 30% (*v*/*v*) acetic acid (250 μL) to the mixture and followed by measuring the trypsin activity at an absorbance of λ = 410 nm using a spectrophotometer (UV-1601, Shimadzu, Kyoto, Japan). One unit of activity was defined as 1 μmol of ρ-nitroaniline released per min and calculated with the following equation [40]:

$$\mathrm{Trypsin}\;\mathrm{activity}\;\left(\mathrm{unit}/\mathrm{mL}\right)=\frac{\mathrm{Absorbance}\;410\;\mathrm{nm}\;\times 1000\times \;\mathrm{mixture}\;\mathrm{volume}\;\left(\mathrm{mL}\right)}{8800\times \;\mathrm{reaction}\;\mathrm{time}\;\left(\min\right)\times 0.025}$$

where 8800 (cm^−1^ M^−1^) is the molar extinction coefficient of ρ-nitroaniline measured at λ = 410 nm.

### 2.5. Protein Assay

The concentration of protein in both ICEs was determined at λ = 750 nm by using BSA (1 mg mL^−1^ as a standard) and Folin–Ciocalteau reagent according to the Lowry et al., 1951 [41] method.

### 2.6. Characterization of Trypsin by SDS-PAGE Electrophoresis

SDS-PAGE electrophoresis was performed to determine the protein pattern in ICEs from both sturgeon species [42]. Each ICE was mixed at 2:1 (*v*/*v*) ratio with sample buffer (62.5 mM Tris–HCl pH 6.8, 2% SDS (*w*/*v*), 10% (*v*/*v*) glycerol, 0.3% (*w*/*v*) bromophenol blue and 5% (*v*/*v*) ß-mercaptoethanol) and boiled for 10 min. Thereafter, the ICEs (with protein concentration of 15 µg) were loaded onto the gel made of 4% stacking gel and 12% separating gel and the electrophoresis was run at a constant current of 15 mA using a vertical electrophoresis system (Bio-Rad Laboratories, Inc., Hercules, CA, USA). After the run, protein bands present in the gel were stained with 0.1% Coomassie Brilliant Blue (G-250) in methanol (35%) and acetic acid (7.5%) and unstained in methanol (35%) and acetic acid (7.5%).

Casein-zymography was performed after electrophoresis for detection of proteases in both ICEs as described by Garcia-Carreno et al., 1993 [43]. Both ICEs were submitted to native-PAGE electrophoresis in a same manner of SDS-PAGE where samples were not boiled, and SDS and reducing agent were removed. After the run, the gel was immersed in 50 mL of a casein solution (20 mg mL^−1^ in 50 mM Tris–HCl, pH 7.5) for 1 h at 4 °C with gentle agitation to allow diffusion of the casein into the gel. Thereafter, the gel was transferred to another solution (50 mL) containing casein (20 mg mL^−1^ in 50 mM Tris–HCl, pH 8.5, 10 mM CaCl_2_) for 20 min at 55 °C with continuous agitation. The gel was then stained with 0.1% Coomassie Brilliant Blue (R-250) in methanol (35%) and acetic acid (7.5%) and unstained in methanol (35%) and acetic acid (7.5%). The presence of proteolytic activities in both ICEs was indicated by the appearance of clear zones on the blue background of the gel, which meant that casein was digested by the targeted protease in these areas.

To reveal the trypsin present in both ICEs, the inhibitory activity staining was used after the submission of both ICEs to native-PAGE electrophoresis as described by Ahmad and Benjakul [44] with a slight modification. After the run, the gel was immersed in 30 mL of an SBTI solution (1 mg mL^−1^ in 50 mM Tris–HCl, pH 8.5, 10 mM CaCl_2_) for 30 min at 4 °C to allow diffusion of SBTI into the gel. Thereafter, the incubation of gel was performed for 40 min at 55 °C and followed by washing in cold distilled water and staining with 0.05% Coomassie Brilliant Blue (R-250) to appear inhibitory zones, indicating the presence of the trypsin in both ICEs. The molecular weight of the trypsin that appeared in both ICEs was estimated using wide-range molecular weight markers (PM2700, SMOBIO, Hsinchu, Taiwan) by calculating the trypsin Rf in comparison with those of protein markers.

### 2.7. Optimum Temperature and Thermostability

To determine the optimum temperature for trypsin activity, the activity of this alkaline protease was measured in both ICEs at different temperatures, including 10, 25, 35, 45, 50, 55, 60, 65, and 70 °C after 20 min of incubation at pH 8.5, using 1 mM BAPNA as a substrate. For the thermostability test, both ICEs were incubated at the above-mentioned temperatures for 30 min and then cooled in an ice bath for assay of residual activity of the enzyme at pH 8.5 as described by Zamani et al., 2014 [40].

### 2.8. Optimum pH and Stability

Different buffers in the pH range of 4.0 to 11.0 (50 mM acetic acid–sodium acetate for pHs 4–6; 50 mM Tris–HCl for pHs 7–9 and 50 mM glycine–NaOH for pHs 10–11) were used for determining the optimum pH for trypsin activity. Both ICEs were used, and they were incubated using1 mM BAPNA as a substrate after 20 min of incubation at 55 °C at different pHs. For the pH stability test, the remaining activity of the trypsin from each ICE was measured using 1 mM BAPNA as a substrate at 55 °C after being incubated at the above-mentioned pHs for 30 min [40].

### 2.9. Effect of Inhibitors

Several protease inhibitors (0.01 mM pepstatin A, 0.05 mM SBTI, 1 mM iodoacetic acid, 2 mM EDTA, 5 mM TLCK, 5 mM TPCK, 5 mM ß-mercaptoethanol, and 10 mM PMSF) were prepared in the relevant solvents and incubated with an equal volume of each ICE at room temperature for 15 min. The remaining activity of the enzyme was then measured by 1 mM BAPNA as a substrate (at 55 °C, pH 8.5) and the percent inhibition was calculated according to the method of Khantaphant and Benjakul, 2010 [45]. The trypsin activity of control was measured in the same manner without the presence of inhibitors and scored to 100%. 

### 2.10. Effect of Metal Ions

To investigate the effect of metal ions (5 mM) on the trypsin activity of both ICEs, univalent (K^+^, Na^+^) and divalent (Ca^2+^, Cu^2+^, Zn^2+^ and Co^2+^) cations were dissolved in 50 mM Tris–HCl (pH 8.5) and then incubated with an equal volume of each ICE for 30 min at room temperature. The residual activity of the enzyme was determined using 1 mM BAPNA as a substrate at 55 °C and pH 8.5 [40]. The enzymatic activity of control was assayed without the presence of metal ions and taken as 100%.

### 2.11. Effect of Surfactants and Oxidizing Agents 

The effect of surfactants (anionic: SDS and sodium cholate; non-ionic: saponin and Triton X-100, all at 1%) and oxidizing agents (sodium perborate at a concentration of 1% and H_2_O_2_ at three concentrations of 5%, 10%, and 15%) on the trypsin activity was measured by incubation of the above-mentioned surfactants and oxidizing agents with an equal volume of each ICE for 1 h at 40 °C. The residual activity of the enzyme was then determined at 55 °C and pH 8.5 using 1 mM BAPNA as a substrate. The assessment of control enzymatic activity was conducted in a similar condition in the absence of chemicals and scored to 100% [25].

### 2.12. Statistical Analysis

This study was conducted on the basis of a completely randomized design, and a one-way ANOVA was used for data analysis using SPSS package 22.0 (SPSS Inc., Chicago, IL, USA). All experimental assessments were performed in triplicate, and data was expressed as the mean ± standard deviation (SD). The comparison of means was carried out by Duncan’s multiple range tests with a statistical significance at *p* < 0.05.

## 3. Results and Discussion

Fish proteases such as trypsin have been the main objective of many of studies, but it is difficult to compare the results obtained in different species because the data are affected by the use of many different methodologies, the state of feeding of experimental animals (fed vs. starved fish), and the type of enzyme preparation (intestinal tissues alone vs. intestinal extracts with the intestinal content) [46].

### 3.1. Protein Pattern, Casein-Zymographyand Inhibitory Activity 

**Figure 1:**
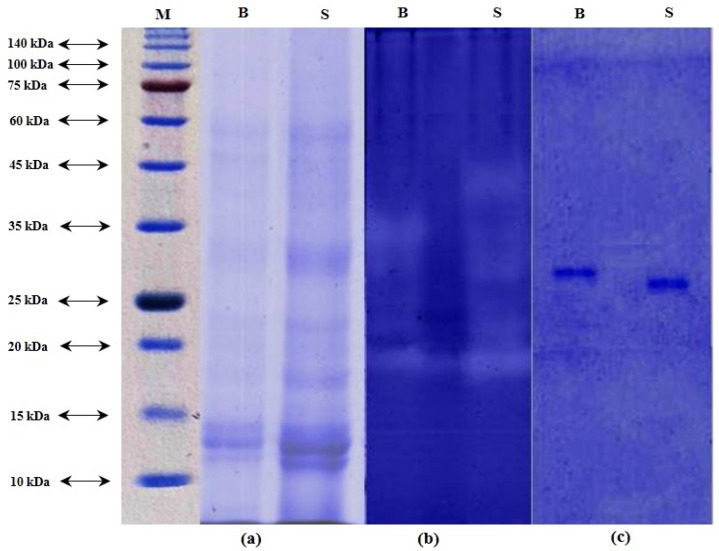
SDS–PAGE (**a**), zymography (**b**), and inhibitory activity staining (**c**) of the intestinal crude extract (ICE) of *Huso huso* and *Acipenser stellatus*. (**a**): Molecular weight marker (M); ICE from *Huso huso* intestine (B); ICE from *Acipenser stellatus* intestine (S). Coomassie blue G-250 (0.1%) was used for staining proteins from SDS-PAGE. Zymogram activity and inhibitory activity staining were stained by using 0.1% Coomassie blue R-250.

Protein pattern of ICE from beluga and sevruga is depicted in Figure 1a. Based on results obtained from the SDS-PAGE, a number of proteins with different molecular weights were shown in the ICE of both sturgeon species. The major bands of each ICE appeared between molecular weights comprised between 10 and 60 kDa.

Casein-zymogram can be used as a highly sensitive and fast assay method for detecting nanograms of protein. The protease activity of both ICEs was demonstrated by casein-zymography as illustrated in Figure 1b. The clear bands, showing the presence of protease, appeared on the gel with different molecular weights. Based on the zymogram pattern, proteases present in ICE from beluga were observed in the range of molecular weights between 19 to 35 kDa, while those in the ICE of sevruga ranged from molecular weights of 19 to 45 kDa.

Inhibitory activity staining for detection of trypsin in ICEs is depicted in Figure 1c. Results showed that a single band for each ICE clearly appeared on the gel with a molecular weight of 27.5 and 29.5 kDa for sevruga and beluga, respectively. In general, trypsins have molecular weights in the range of 20–30 kDa [47]. In particular, different molecular weights for trypsins have been reported in various fish species such as 21.7 kDa for mrigal carp [48], 23.2 kDa for common kilka [40], 23.5 kDa for pirarucu [49], 24 kDa for small red scorpion fish [50], 21 and 24 kDa for liver of albacore tuna [51], 24 kDa for catfish [52], 24.4 kDa for gulf corvina [53], 25 kDa for Monterey sardine [54], 26 kDa for common dolphinfish [55], 27 kDa for zebra blenny [56], 28.8 kDa for sardinelle [57], 29 kDa for Atlantic bonito [58], 38.5 kDa for tambaqui [59], and 42 kDa for skipjack tuna [60]. However, several reasons such as different habitat and climate, autolytic degradation, and genetic variation among fish species may explain why trypsins from various sources have different molecular weights [60,61].

### 3.2. Optimum Temperature and Thermostability

**Figure 2:**
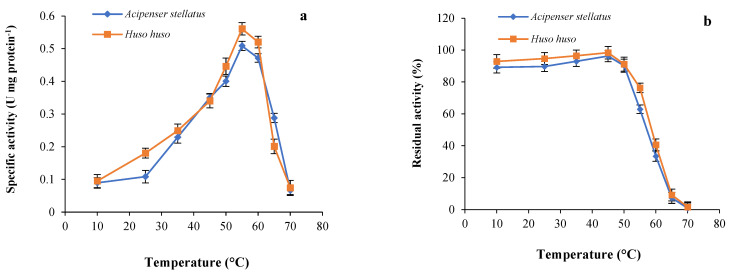
(**a**) Optimum temperature: the enzymatic activity of both trypsins was measured at different temperatures at pH 8.5 using BAPNA as a substrate; (**b**) thermostability: residual activity of both enzymes was determined at 55 °C and pH 8.5 after incubating at different temperatures for 30 min using BAPNA as a substrate.

Enzymes are one of the main biological macromolecules and their maximum activity depends on an optimum temperature to make them functional. Figure 2a revealed that optimum temperature of the trypsin in ICE prepared from beluga and sevruga was found to be 55 °C, although 92.70% of the maximum activity of the enzyme was still maintained at 60 °C for both sturgeon species. However, an obvious decrease in the trypsin activity of both ICEs was observed at temperatures above 60 °C, probably due to thermal inactivation of this enzyme caused by protein unfolding [40]. Similar optimum temperature (55 °C) was recorded for trypsins in skipjack tuna [26], gilthead seabream [46], sardinelle [57], and silver mojarra [62]. Optimum temperature of trypsin for both sturgeon species was higher than values reported for cold-water fish such as Atlantic cod (*Gadus morhua*) [63], grey triggerfish [25], lane snapper (*Lutjanus synagris*) [64], and Japanese sea bass (*L. japonicus*) [65] indicating optimum temperatures over a range of 40–45 °C. These differences could be attributed to the temperature of the fish habitat or experimental conditions used in assessments [66]. The optimum temperature for enzyme maximum activity may be interesting for comparative physiological studies, even though such data offer limited information on enzyme activity under normal rearing conditions. Although fish trypsins are mostly unstable at temperatures higher than 40–50 °C, their thermal stability is well known to be at temperatures below 40 °C [67]. Trypsin thermal stability from ICE of beluga and sevruga is displayed in Figure 2b. As can be observed in this figure, the stability of both trypsins was highly maintained up to 50 °C with a remaining activity of 90.2% and 91.7% for sevruga and beluga, respectively. A gradual decrease in the activity of both trypsins was recorded at 55 °C, whereas enzymatic activity sharply decreased at 60 °C. After heating the ICEs at 70 °C, the relative activities for both trypsins were only about 0.9% and 1.6% of their initial activity for sevruga and beluga, respectively. These results were in accordance with those of sardinelle, common kilka, mrigal carp, and pirarucu, which were exhibited to be stable up to 50 °C [40,48,49,57]. The trypsins from beluga and sevruga showed to be more stable at high temperatures in comparison with those reported for the Monterey sardine, chinook salmon, bluefish, Tunisian barbell, and common dolphinfish that the enzymatic activity was rapidly lost at temperatures above 40 °C [54,55,58,68,69]. In general, thermostability of the trypsin enzyme might vary by some factors such as fish species and experimental conditions [23,70]. From a biological point of view, it is difficult to deduce any advantage for beluga and sevruga in possessing proteases showing different resistances to heating, since the normal temperature of water rarely exceeds 21–24 °C. Nevertheless, from a biotechnological perspective, it may be interesting to have information about active and easily denaturalizable proteases potentially useful in the feed industry [71].

### 3.3. Effect of pH on Trypsin Activity and Stability

**Figure 3:**
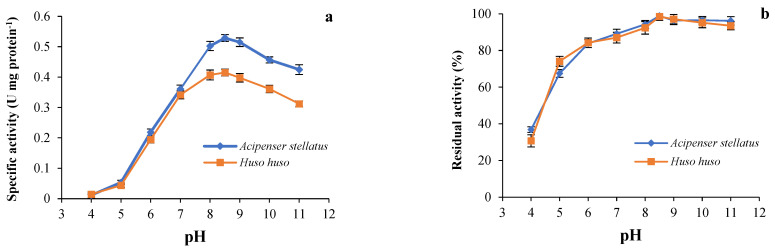
(**a**) Optimum pH: the enzymatic activity of both trypsins was determined at different pHs at 55 °C using BAPNA as a substrate; (**b**) pH stability: residual activity of both enzymes was measured at 55 °C and pH 8.5 after incubating at different pHs for 30 min using BAPNA as substrate.

The results observed from the effect of pH on the activity and stability of trypsin from beluga and sevruga are illustrated in Figure 3. Trypsins from both species had a maximal activity at pH 8.5 (Figure 3a). Trypsin activity was dramatically reduced at pH values ranging from 4.0 to 5.0. Our results showed that the stability of trypsin from both sturgeon species was highly preserved at pH values comprised between 5.0 and 11.0 with activity values of 75% for beluga and 80% for sevruga. The high ranges of pH may change the net charge and conformation of an enzyme and inhibit to bind to the substrate properly, resulting in the abrupt loss of enzymatic activity [60,72]. Trypsins are mainly known to have more activity within a range of pH values comprised between 7.5 and 10.5 [46,73]. The optimum pH (8.5) recorded for trypsin in both sturgeon was similar with results reported for trypsins from the brownstripe red snapper viscera and the albacore tuna hepatopancreas [45,51], whereas both trypsins indicated the lower optimum pH than those recorded for the Japanese sea bass, gilthead seabream and common dentex, and the pirarucu [46,49,65]. However, optimum pH may differ depending upon the experimental conditions such as concentration and type of substrate, temperature, and type of metal ions [60]. For instance, Martinez et al., 1988 [74] showed that the trypsin from pyloric caeca of the anchovy had the optimum pHs of 8.0 and 9.5 for the hydrolysis of BAPNA and casein, respectively.

The effect of pH on the trypsin stability in both ICEs is displayed in Figure 3b. The stability of both enzymes was considerably retained between pH 6.0 to 11.0. Trypsin activity was lost about 63.15% and 69.26% at pH 4.0 in *sevruga* and beluga, respectively, while the loss of the enzyme activity at pH 5.0 was recorded by 34.13% and 35.84% for *sevruga* and beluga, respectively. However, trypsin in ICE of *sevruga* and beluga lost only 1.19% and 1.51% of its activity at pH 8.5, respectively. Similar behavior was reported for trypsins from common kilka [40], common dolphinfish [55], and zebra blenny [56] which retained 80–100% of the activity at pH ranges of neutral and alkaline. The high catalytic activity of the trypsin is observed in alkaline pHs, and its stability at a particular pH may be linked to the net charge of the enzyme at that pH [75].

### 3.4. Effect of Inhibitors on Trypsin Activity 

The sensitivity of protease enzymes to various inhibitors is a valuable tool for their proper functional characterization [55]. Based on their nature, inhibitors can be classified into two classes: chemical inhibitors and protein inhibitors [76]. A trypsin inhibitor is a type of serine protease inhibitor that reduces the biological activity of trypsin thereby rendering it unavailable to bind with proteins for the digestion process [77,78]. Therefore, it can be considered important to characterize the effect of different inhibitors on the trypsin activity.

Table 1 shows the effect of different inhibitors on the activity of trypsin from beluga and sevruga. As it is shown in this table, a serine protease inhibitor such as PMSF inhibited 39.11% and 36.29% of the trypsin activity in *sevruga* and beluga ICE samples, respectively. Both enzymes were completely inhibited by trypsin specific inhibitors such as SBTI and TLCK, while a chymotrypsin-specific inhibitor (TPCK) did not show any inhibitory effect on their enzymatic activity (*p* > 0.05). Furthermore, a metalloproteinase inhibitor (EDTA) and a disulfide bond reducing agent (ß-mercaptoethanol) had a partial inhibitory effect on trypsin activity in both sturgeon species, although the inhibition rates varied between both species. In particular, trypsin from *sevruga* was inhibited by ß-mercaptoethanol (25.33%) and EDTA (23.55%) more than those in beluga (22.84 and 21.06%, respectively) where no significant difference was shown between both species (*p* > 0.05). Results from EDTA indicate the high dependence of trypsin activity from both sturgeon species on divalent cations [46]. However, an aspartic proteinase inhibitor (Pepstatin A) and a cysteine proteinase inhibitor (iodoacetic acid) exhibited a negligible inhibitory effect on the trypsin activity of both species. Similar results have been observed in other fish species [40,51,56]. For instance, Khangembam and Chakrabarti, 2015 [48] reported that trypsin activity from the digestive system of mrigal carp was inhibited by SBTI and TLCK. SBTI is a single polypeptide chain that acts as a reversible competitive inhibitor of trypsin and forms a stable, enzymatically inactive complex with trypsin, resulting in reduction of the enzyme availability [79]. TLCK is an irreversible inhibitor of trypsin and trypsin-like serine protease that deactivates these enzymes through the formation of a covalent bond with histidine residue in the catalytic site of the enzyme and blocks the active center of the enzyme for binding to substrate [80]. As reported by several authors [40,49,56], PMSF strongly inhibited the activity of trypsin from viscera of common kilka, pirarucu, and zebra blenny, respectively, whereas TPCK had no effect on the enzyme activity from common kilka [40]. The trypsin activity from the intestine of common dolphinfish was partially inhibited by β-mercaptoethanol and EDTA [54], while the trypsin activity from liver of albacore tuna was not reduced in the presence of pepstatin A and iodoacetic acid [51].

### 3.5. Effect of Metal Ions

Metal ions have a key role in the activity regulation of many enzyme-catalyzed reactions [81]. Our results on the effect of metal ions on the trypsin activity in *beluga* and sevruga are detailed in Table 2. No significant effect on the activity of *both enzymes* was found in the presence of univalent cations Na^+^ and K^+^ (*p* > 0.05). The enzymatic activity of trypsin in both species was significantly reduced by divalent cations Cu^2+^, Zn^2+^ and Co^2+^, whereas Ca^2+^ significantly enhanced the activity of both trypsins (*p* < 0.05). Similar results on the effect of Ca^2+^ on the trypsin activity were also observed in common kilka, common dolphinfish, and zebra blenny [40,55,56]. The attachment of Ca^2+^ to the active site of serine proteases such as trypsin not only increases the stability of the enzyme structure, but it also protects the enzyme from self-digestion [66,69,82]. The enzymatic activity of trypsin in common dolphinfish was reduced by 82% and 81% by Zn^2+^ and Cu^2+^, respectively [55], while 100% of enzymatic activity of tryspin from zebra blenny was lost in the presence of Zn^2+^ and Cu^2+^ [56]. In common kilka [40], no inhibition was observed in the trypsin activity in presence of Na^+^ and K^+^. Differences in percent inhibition might be linked to species diversity, environmental adaptations and feeding habits of fish [83].

### 3.6. Effect of Surfactants and Oxidizing Agents 

Surfactants are the most widely used groups of compounds today, with wide application in industry and household. These are unique substances that contain hydrophobic and hydrophilic moieties within their molecule and find enormous applications in biology. Oxidizing agents such as sodium perborate and H_2_O_2_ are also used in the detergent industry as bleaching agent. Surfactants and oxidizing agents may be harmful or even toxic to aquatic organisms. These compounds can penetrate through tissues and bind to biomolecules, such as enzymes, causing changes in cellular activity [84]. As reviewed by Rubingh, 1996 [85], surfactants can influence the activity of enzymes in two ways. Firstly, by binding to the enzyme, surfactants can influence intrinsic enzyme properties such as the secondary and tertiary structure or flexibility, and thereby, affect its ability to serve as a catalyst. A less direct, but equally important, way in which surfactants affect enzyme activity is by changing the environment in which the enzyme functions. It is well-known that SDS disrupts non-covalent bonds within and between enzymes, denaturing them, and resulting in the loss of their native conformation and function [86], whereas saponin, Triton X-100, and sodium cholate are the non-denaturing surfactants [87,88,89]. Hence, characterizing the effect of these chemical compounds on trypsin activity is of relevance for proper characterizing its activity.

The results on the effect of various surfactants and oxidizing agents on the trypsin activity in *sevruga* and beluga are shown in Table 3. A significant increase in the activity of both trypsins was observed after incubation for 1 h at 40 °C in the presence of surfactants tested, including saponin, sodium cholate, and Triton X-100 at final concentrations of 1% (*p* < 0.05). Both trypsins were highly unstable against sodium dodecyl sulfate (SDS), in which trypsins from *sevruga* and beluga significantly lost about 94% and 97% of their activity in the presence of 0.1% SDS, respectively (*p* < 0.05). Similar results were found in trypsins of other fish species in the presence of saponin, sodium cholate, Triton X-100 and SDS [40,56]. The obtained results on the effect of oxidizing agents on both trypsins showed that the enzymatic activity was reduced in the presence of sodium perborate (1%) in sevruga and beluga by 22.23% and 24.37%, respectively. The activity of both enzymes was also decreased significantly with an increase in H_2_O_2_ concentrations from 5% to 15%, as described in Table 3 (*p* < 0.05). Trypsin from sevruga showed significantly higher activity than trypsin from beluga in the presence of H_2_O_2_ ranging from 5% to 15%, indicating that trypsin from sevruga was more tolerant to H_2_O_2_ than trypsin from beluga. The biochemical and structural properties of enzyme can affect its ability as a catalyst in presence of oxidizing agents [85]. These results showed that trypsins from *sevruga* and beluga were more stable against H_2_O_2_ than trypsins from grey triggerfish and zebra blenny [25,56], whereas most proteases have shown to be unstable in the presence of oxidizing agents like hydrogen peroxide [25].

## 4. Conclusions

The results of our study indicated that trypsin from intestine of beluga and sevruga had similar properties to trypsins from bony fish. The enzyme had an optimum temperature of 55 °C and thermal stability was maintained over 90% up to 55 °C. This alkaline protease had an optimum pH of 8.5 and showed to be tolerant in the pH range of 6.0 to 11.0 in both studied sturgeon species. The molecular weight of trypsin for sevruga and beluga was estimated to be 27.5 and 29.5 kDa, respectively, as data from inhibitory activity staining indicated. Both trypsins were inhibited by main specific inhibitors, SBTI and TLCK. Additionally, the enzymatic activity of trypsins was still detected after 1 h in the presence of surfactants and oxidative agents. Information provided in this manuscript related to trypsin activity for beluga and sevruga based on changes in the activity of this alkaline protease based on a tested range of pH and temperature values, and presence of potential inhibitors, ions and cations may be considered a preliminary step to design in vitro tests for the assessment of protein digestibility in these species.

## Figures and Tables

**Table 1:** Effect of various inhibitors on the activity of trypsin from *Huso huso* and *Acipenser stellatus*.

Inhibitors	Concentration	Inhibition (%)	
		*Huso huso*	*Acipenser stellatus*
Control	-	0.0 ± 0.00 ^a^	0.0 ± 0.00 ^a^
PMSF	10 mM	36.29 ± 0.39 ^c^	39.111 ± 0.45 ^c^
SBTI	0.05 mM	99.81 ± 0.54 ^d^	99.29 ± 0.49 ^d^
TLCK	5 mM	99.19 ± 0.57 ^d^	99.51 ± 0.47 ^d^
TPCK	5 mM	0.00 ± 0.00 ^a^	0.00 ± 0.00 ^a^
Pepstatin A	0.01 mM	0.51 ± 0.003 ^a^	0.89 ± 0.002 ^a^
Iodoacetic acid	1 mM	0.25 ± 0.002 ^a^	0.44 ± 0.003 ^a^
EDTA	2 mM	21.06 ± 0.36 ^b^	23.55 ± 0.51 ^b^
ß-Mercaptoethanol	5 mM	22.84 ± 0.41 ^b^	25.33 ± 0.37 ^b^

Each intestinal crude extract (ICE) was incubated with same volume of inhibitor at room temperature for 15 min and the enzyme activity was determined at pH 8.5 and 55 °C. The control was prepared with similar conditions in absence of inhibitors. Data were represented by mean ± standard deviation and compared by Duncan’s multiple-range test. Different superscripts in the same column show statistical difference while there is no significant difference in the same row (*p* < 0.05).

**Table 2:** Effect of various metal ions on activity of trypsin from *Huso huso* and *Acipenser stellatus*.

Metal Ions	Concentration	Residual Activity (%)	
		*Huso huso*	*Acipenser stellatus*
Control	-	100 ± 0.00 ^d^	100 ± 0.00 ^d^
KCl	5 mM	99.6 ± 1.05 ^d^	99.2 ± 0.92 ^d^
NaCl	5 mM	99.3 ± 1.03 ^d^	99.5 ± 1.01 ^d^
CaCl_2_	5 mM	107.21 ± 0.81 ^e^	105.32 ± 0.98 ^e^
CuCl_2_	5 mM	48.31 ± 0.88 ^a^	44.58 ± 0.96 ^a^
ZnCl_2_	5 mM	67.79 ± 1.15 ^b^	64.83 ± 1.23 ^b^
CoCl_2_	5 mM	76.69 ± 0.85 ^c^	74.77 ± 1.05 ^c^

Univalent and divalent metal ions were incubated with same volume of each intestinal crude extract (ICE) at room temperature for 30 min and residual activity of the enzyme was measured at pH 8.5 and 55 °C. The control was prepared with similar conditions without metal ions and scored as 100%. Data were represented by mean ± standard deviation and compared by Duncan’s multiple-range test. Different superscripts in the same column show statistical difference while there is no significant difference in the same row (*p* < 0.05).

**Table 3:** Trypsin activity from *Huso huso* and *Acipenser stellatus* in presence of surfactants and oxidizing agents.

Chemicals		Concentration	Residual Activity %	
			*Huso huso*	*Acipenser stellatus*
Surfactants	Control	-	100 ± 0.00 A ^d^	100 ± 0.00 A ^d^
	Triton X-100	1%	108.81 ± 1.62 A ^e^	112.55 ± 1.11 A ^e^
	SDS	1%	2.79 ± 0.21 A ^a^	5.77 ± 0.33 B ^a^
	Sodium cholate	1%	132.23 ± 1.12 A ^j^	197.77 ± 0.99 B ^j^
	Saponin	1%	126.14 ± 1.06 A ^f^	128.44 ± 1.46 A ^f^
Oxidising agents	Sodium perborate	1%	75.63 ± 1.07 A ^c^	77.77 ± 0.97 A ^b^
	H_2_O_2_	5%	70.30 ± 1.02 A ^c^	87.55 ± 1.02 B ^c^
		10%	64.97 ± 1.19 A ^c^	84.55 ± 0.73 B ^c^
		15%	40.35 ± 0.65 A ^b^	73.77 ± 0.91 B ^b^

Each intestinal crude extract (ICE) was incubated with same volume of the chemicals for 1 h at 40 °C and residual activity of the enzyme was measured at pH 8.5 and 55 °C. The control was prepared with similar conditions without chemicals and scored as 100%. Data were represented by mean ± standard deviation and compared by Duncan’s multiple-range test. Different capital letters in the same row and different superscripts in the same column show statistical difference (*p* < 0.05).

## Data Availability

Not applicable.
